# After the handover: Exploring MSF’s role in the provision of health care to migrant farm workers in Musina, South Africa

**DOI:** 10.1080/17441692.2019.1586976

**Published:** 2019-03-05

**Authors:** Thea de Gruchy, Anuj Kapilashrami

**Affiliations:** a The African Centre for Migration & Society, University of the Witwatersrand, Johannesburg, South Africa; b Centre for Global Public Health, Queen Mary University (London), London, UK

**Keywords:** Migration, migration and health, critical humanitarianism, South Africa, Médecins sans Frontières

## Abstract

Non-state actors, including humanitarian agencies, play a prominent role in providing health care in low- and middle-income countries. Between 2007 and 2009, Musina, a South African municipality bordering Zimbabwe, became the site of several interventions by non-state organisations as an unprecedented number of Zimbabweans crossed the border, putting strain on already burdened local systems. After the initial need for humanitarian relief dissipated, organisations started to implement projects that were more developmental in nature. For example, Médecins sans Frontières developed a mobile clinic programme to improve health care access for migrant farm workers, a programme that was subsequently integrated into the Department of Health. Since the handover of the programme, it has faced multiple challenges. Using qualitative methodology and a case study approach, this paper traces the development of the programme, exploring the changing relationship between MSF and the state during this time. This research raises questions about the implications of short-term ‘innovative’ interventions targeting the access that migrants have to care, within a context in which policy and programmatic responses to health are not migration aware. Furthermore, it highlights the ways in which the energies and resources of local DoH employees were redirected by MSF's involvement in the area.

## Introduction

Within South Africa, as in many low- and middle-income countries, the provision of health care by the state is often accompanied by non-state intervention, specifically in the form of non-governmental organisations. These interventions are usually portrayed as either humanitarian, in response to ‘disruptions and emergencies, such as war, famine and natural disasters, with an emphasis on the short term’ (Crewe & Axelby, 2013, pp. 3, 4) or developmental, where the idea is ‘to produce long-term social, economic or political change’ (Crewe & Axelby, 2013, pp. 3, 4). Médecins sans Frontières (MSF), the organisation at the centre of this paper, have traditionally engaged in work that is more humanitarian in nature. However, the boundaries between the two sectors are increasingly blurring with shifts in broader discourses underpinning their approaches. As Crewe and Axelby (2013) argue, ‘development usually involves some shorter-term aid – helping the poorest families out when they are destitute – and humanitarian aid often aims for longer-term service provision’ (pp. 3, 4).

One of the key critiques of development initiatives tied to international funding has been around the ways in which they disrupt both local state structures and civil society. Scholars have highlighted the results of disparities in salaries and working and living environments between agency staff and local civil servants (Pfeiffer, 2003) and the ways in which public infrastructure is weakened by state employees taking up better paying jobs and opportunities with international non-governmental organisations (iNGOs) (Kapilashrami & McPake, 2013). Empirical research has also shown how larger iNGOs can detract attention from smaller local organisations (Bruno-van Vijfeijken, Lux, Neupane, & Singh, 2017) and how international initiatives, like the Global Fund through, what Kapilashrami and O’Brien refer to as, their ‘dictating regime’ (2012) shape the agendas of both local civil society and the state. Through funding cycles and associated conditions, specific kinds of work and approaches are prioritised (Younis, 2007), and attendance at trainings and workshops tied to specific projects and their deliverables is demanded (Kapilashrami & O’Brien, 2012; Pfeiffer, 2003).

This critique has primarily focused on interventions linked to development and health aid. Critiques of humanitarian aid, on the other hand, have traditionally been far more concerned with the relationship between the organisations and their ‘expatriate’ and ‘local’ workforces (Redfield, 2012, 2013), as well as these organisations’ changing understanding of themselves and their duty to interfere, during what is perceived to be a humanitarian crisis (Fassin, 2010; Fox & Goemaere, 2006). However, as the narrative around what constitutes a ‘crisis’ has changed, specifically in relation to HIV/AIDS and migration, the form and substance of humanitarian interventions have started to change. Within the South African context, for example, MSF has maintained a presence and provided HIV and TB care in Khayelitsha, an informal township in Cape Town, since 2001, and has a secondary aim of ‘influencing local, national, regional and international policies on treatment for these diseases’ (Médecins Sans Frontières, n.d.). Understanding the longer term implications of these interventions, traditionally the interest of critiques of development interventions, is therefore imperative.

Using a case study of MSF's involvement in Musina, a South African town bordering Zimbabwe, this paper traces the development and sustainability of the programme and looks at the longer-term implications of MSF's intervention for the local Department of Health (DoH). This case study explores the changing relationship between MSF and the state during this time. It highlights the ways in which the energies and resources of local DoH employees were redirected by MSF's involvement in the area, and interrogates how this has changed since MSF's departure, specifically in light of the different approaches that MSF and the DoH have taken in the management of the programme. Furthermore, it raises important questions about the exceptionalisation of migrants through such targeted programmatic interventions.

## Migration and health in Musina: Before and after ‘crisis’

Bordering Zimbabwe, Vhembe District Municipality is a space in which cross-border migrants are particularly visible, specifically in the town of Musina. Between 2007 and 2009, the number of Zimbabweans crossing the border and converging on the town peaked due to electoral violence and a widespread cholera outbreak, putting a strain on local systems and structures. In December 2008, Vhembe District was declared a disaster area in the wake of a cholera outbreak in Musina and surrounding areas (Vearey & Anderson, n.d.). In response, many international organisations, including MSF, the International Organization for Migration (IOM), the United Nations High Commissioner for Refugees (UNHCR), and Save the Children UK, moved into the area to respond to ‘the coalface of humanitarian crisis’ (Rondganger, 2008). In this period, these organisations focused on providing emergency health care, assisting with documentation, and monitoring the detention and deportation of immigrants.

Once the initial ‘crisis’ was over, as the number of Zimbabweans steadied and then decreased in Musina, many of these organisations remained, with a renewed focus on longer-term concerns. MSF shifted its focus from providing access to emergency medical care to improving the access that migrant farm workers around the town had to general and comprehensive health care, through putting in place mechanisms which could be maintained by local structures once the organisation left. This resonates with Steinberg's reflections on MSF's intervention in Lusikisiki, on the South African coast, about which he writes ‘it's goal … was to show that ordinary nurses, not rich international NGOs, could administer a treatment programme’ (Steinberg, 2008, p. 87).

Whilst South Africa has a functioning public health care system, this system is faced with the ‘so-called four colliding epidemics’ – HIV and tuberculosis (TB); injury and violence; chronic illness and mental health; and maternal, neonatal, and child health (Mayosi et al., 2012). Furthermore, until 2009, state policy was characterised by HIV/AIDS denialism, which provided ample room for actors like MSF to engage in both advocacy and health care provision (Redfield, 2013, p. 246). Since 2009, the state has made strides in rectifying its previous position on HIV. However, South Africa still has a high incidence rate for both TB and HIV; violence remains rife (Mayosi et al., 2012); and the burden of non-communicable diseases in the country is growing (Mayosi et al., 2009). Furthermore, health policy within South Africa (and across the region) has failed to account for the realities of migration and recognise migration as a determinant of health (Vearey, 2014; Vearey, Modisenyane, & Hunter-Adams, 2017). Migrants, both cross-border and internal, face barriers to care, as systems do not take into account their mobility and temporary nature of residence, including the need for patients to be able to access medication, particularly chronic medication, as they move for work, due to ill-health, or simply return home for Christmas (Vearey et al., 2017). For cross-border migrants, access can be further undermined by their treatment at the hands of frontline staff at health care facilities, who often deny or delay care to foreign patients, verbally abuse and incorrectly charge them (Makandwa & Vearey, 2017; Vearey, 2014).

In addition, since the mid-1990s, the DoH has been restructured to reflect a decentralised approach to governance and administration. Decentralisation, described by McIntyre and Klugman as ‘the transfer of responsibility for planning, management and financing from central government to peripheral levels of government’ has been a key approach in health sector reform in South Africa, as in other low- and middle-income countries over the past decade or more (2003, p. 109). However, within the South African DoH, decentralisation has been deemed ineffective and has invoked strong feelings of resentment and disempowerment among civil servants at district and facility level as staff are expected to implement policies, with very little forewarning and insufficient resources, but remain excluded from decision-making processes (McIntyre & Klugman, 2003; Walker & Gilson, 2004).

Within this context, there have been many opportunities for non-state actors to engage with the provision of health care and advocate for improved access – opportunities which, at various points, MSF has taken. Against this backdrop, this paper investigates the changing relationship and dynamics between MSF and the state agencies, and the implications of this since MSF's departure from Musina in 2013.

## Methods

This paper forms part of a larger qualitative study of the programmatic and policy responses to migration and health in Musina. This paper focuses on the development of a MSF-led mobile clinic programme, the Musina Model of Care, and uses it as a lens through which to understand the relationship between MSF and the state.

Findings reported in this paper draw on fieldwork conducted between January and November 2017 (with some initial scoping work having been done in 2016, and some follow up being done in 2018) on two farms in the area around Musina, in Musina itself, in Polokwane (the Provincial capital), in Pretoria and Cape Town, and over Skype. This paper draws on data from 79 key informant interviews (see Table 1), published and grey literature collected during the research, and observation of the programme.

**Table 1.** Key informant interviews.

Category of respondent (alphabetical)	Number of interviews
Agricultural sector organisation	5
Civil servants (not DoH Musina)	5
Community Health Workers (CHWs)	3
DoH Musina	4
Farm management	5
Farm workers	43
Local NGOs	4
Médecins sans Frontières (MSF) (current and former employees)	5
Other	5
Total	79

The grey literature, described by Adams *et al.* as second and third tier literature (Adams, Smart, & Huff, 2017), consulted for this study included emails, memos, and project reports that provided information about the projects, and insights into the relationship between MSF and the DoH. These were analysed in relation to published literature pertaining to MSF, as well as iNGOs more generally, and literature about the structure of the South African state, specifically in relation to the health care system.

Observation of the mobile clinic programme was undertaken between May and July 2017. Given the nature of work in the mobile clinics, conducting in-depth interviews with services providers in the programme was difficult and obstructed their work. As such, observation and informal conversations with staff recorded as field notes were used to engage with questions around the continuity of the programme since the departure of MSF. No treatment or care itself was observed.

Analysis of this data was undertaken using thematic analysis, the identification of codes and themes through a reading and examination of the data (Fereday & Muir-Cochrane, 2006). The emergent themes which have shaped this paper include the relationship between MSF and the DoH mobile clinic employees, experiences of providing care to migrant farm workers, and MSF and the DoH's perceptions of the Musina Model of Care and its importance.

Ethical clearance was obtained from the University of the Witwatersrand Non-Medical Research Ethics Committee (REC), protocol H16/08/10. In line with the commitments laid out for the REC, informed consent was sought prior to interviews, observation, and access to grey literature.

In the following sections, we lay out and explore the development of the Musina Model of Care and MSF's involvement in Musina. We present this narrative chronologically in the Results section, for ease of understanding and to highlight the ways in which the relationship between the state-run mobile clinic and MSF changed. We conclude the paper by discussing the broader implications of our findings for migrants’ health and for our understandings of humanitarian interventions.

## Results

### Why healthcare for migrant farm workers?

Once the initial ‘crisis’ in Musina had passed, MSF looked for other ways in which they could intervene in the area. In 2009, the organisation decided to improve the access that migrant farm workers, living and working, either temporarily or more permanently, on the commercial farms in the area surrounding Musina had to health care.

Access to health care for migrant workers, who have crossed the border into South Africa since the 1970s and found work on the commercial farms in the area (Bolt, 2016; Rutherford, 2008a), has traditionally been very limited (Médecins Sans Frontières, 2012, 2013). Research by IOM indicated that at this time, HIV prevalence within these communities was as high as 28.1%, nearly double the prevalence of Vhembe District – 14.7% (International Organisation for Migration, 2010, p. 9). Many workers lived, and continue to live, in ‘compounds’ on the farms, accommodation provided by farm management, which are often 50 kilometres from the nearest healthcare facility. Compounds are characterised by poorly ventilated accommodation, and limited and makeshift sanitation facilities. Furthermore, the geographical isolation of workers on these compounds means that access to healthcare facilities is restricted as it usually involves taking a day off work, thus losing a day's wage and incurring travel costs.^1^ Should blood tests or chronic medication be needed, this would require frequent trips adding to the cost. For Zimbabwean workers, there is the additional stress of having to navigate roaming military and police personnel along the border. Although some do have the relevant documentation, this does not necessarily protect them from harassment or deportation (Rutherford, 2008b). As one farm worker reflected in response to questions about seeking health care prior to the involvement of MSF in the area:

People had to look for money, to enable them to take a ride in cars that go to Musina, and so this meant that if you had no money this would take you some time to access health services. (CA_LM_102)

Prior to the arrival of MSF, the DoH had provided some care to the worker communities by way of a mobile clinic. However, until the intervention of MSF, this mobile clinic programme, which was meant to visit farms on rotation, was not accredited to provide antiretroviral therapy (ART). Furthermore, there was little awareness among the farm workers about the programme and its purpose:

Their vehicle used to come here and we saw it, but we did not know what were they here for … It would come and be parked here for about two hours … and then you would see it leaving, without anyone having accessed health services. (CA_EG_203)

## Developing the Musina model of care

Acknowledging the limitations described above, and wanting to address the particular needs of the migrant farming community, MSF established their own mobile clinic programme, which they called the Musina Model of Care. This was described by MSF as a project through which they could demonstrate ‘innovation’, which they hoped would lead to changes in the ways in which the DoH made care accessible to migrant farm workers specifically, but rural population groups more generally (iNGO_01). As such, the MSF clinic ran parallel to the DoH one but differed from the state programme in three respects.

First, the programme extended the scope of care provided to farm workers by providing HIV testing, counselling, and ART,^2^ by most accounts being the first mobile clinic programme in South Africa to do so. At this time, the state-run mobile clinic provided antenatal, maternal, and child health care, as well as TB treatment. To be tested for HIV or initiated on ART, farm workers had to make the trip to one of the facilities in Musina.

Second, MSF argued that the practices adopted by the Musina Model of Care were innovative and took into account the realities of migration and its affect on continuity of care, a feature lacking from state responses:

Patients were provided with a patient-held health record and asked about their travel plans at each visit. If they were returning to Zimbabwe, MSF provided information about ART sites nearby. Those planning to travel for more than two weeks were classified as a temporary transfer out (TTFO) and were given a three month supply of antiretroviral therapy (ART), one week of tail protection (a strategy to prevent resistance if treatment interruption is unavoidable), and a transfer letter. Counselling materials were designed to help patients understand possible changes in regimen and formulation over time. (Médecins Sans Frontières, 2013, p. 7)

Government health care providers and farm workers all confirmed these aspects of the programme.

Third, as part of their response, MSF asked compound residents to identify individuals who they felt would best perform the duties of a full-time Community Health Worker (CHW) (iNGO_06). As members of the compound community, CHWs were envisaged as acting as a ‘vital link between the farm community and the mobile PHC nurses’, testing for HIV, providing counselling for HIV and TB, promoting awareness, and facilitating support groups for workers on ART (Médecins Sans Frontières, 2013, p. 8). Ten individuals, situated either within a large compound or in an area where several compounds adjoined, were trained and paid a stipend by MSF to act as a link between the mobile clinic nurses and the workers, and insure that basic health care was still accessible between clinic visits (Médecins Sans Frontières, 2013). Support groups, or patient adherence groups would meet on Sundays, and, while they were run by CHWs, were often attended by nurses from the mobile clinic. Findings from a study of support groups attached to the MSF project in Khayelitsha found that these groups lead to ‘improved retention on ART and decreased rates of virologic rebound’ (Luque-Fernandez et al., 2013, p. 4). As in Khayelitsa, interview data showed that this arrangement worked for both workers, who no longer needed to take time off work to collect their medication, and for nurses, who felt that they are able to offer a better service to their HIV positive patients and who were able to claim overtime.

### Working with the DoH

The implementation of this programme and MSF's presence in Musina was initially met with suspicion and hesitation from staff implementing the state mobile clinic programme. The manager of the state programme reported both an unease among the state employed health care providers towards a ‘competing’ programme, but also frustration with the Provincial administration and MSF for not managing the process better:

You know that change is pain … my staff members, they thought that MSF is here to take their jobs … and their (MSF's) attitudes towards the sub-district, it was not an easy thing. Because, people, they did not understand much. Because seemingly the MOU (Memorandum of Understanding signed between MSF and the state) was just from the Province down to the ground, not even involving other people around. And they (MSF) did not establish themselves when they arrived here; they did not knock on the door to see who's here (providing services in the area), they just pushed to the farms. So the relationship at first was not … OK. I had to mediate between the two, so that each party understands that there's nobody here to take another person's job, it's just to take the services to the patients, and MSF is here temporary; they are going and we’re the custodians of the project. (DOH_01)

MSF's project co-ordinator, on the other hand, was critical of the state in which they found the DoH programme. She referred to absenteeism amongst the nurses, a ‘problem with team dynamic’, the team being ‘under resourced in just about every sense’, and a disinterest and lack of motivation on the part of the health care providers, specifically in relation to treating foreign patients (iNGO_03).

However, MSF's involvement in Musina was only short term. To ensure the longevity of the Musina Model of Care, and its innovative approach to health care access for migrant farm workers, MSF started working with the DoH mobile clinic programme in 2010. A series of workshops, trainings, and meetings were thus held between the two teams, who managed to start working together. This included, in 2011, MSF paying for ten registered nurses to partake in a DoH accredited sexual assault nursing course.^3^ A former MSF employee describes this process:

You know when you’re starting anything at first there's not that trust or that fear of the unknown, and then you’ll be like ‘OK, who are these guys?’. I think now we’re working together, the team like every Friday we’ll have like refresher courses. So we’ll include both the Department and MSF, and we start interacting and getting to know each other so when we go to the field it's more easier than you guys just coming in your car and the MSF guys and then we meet in the field. It's a little bit awkward  …  so it started from, I wouldn't say it was smooth sailing ja [yes], but slowly, slowly we became a team. (iNGO_07)

The impetus for the coming together of the two programmes is described slightly differently by the DoH programme co-ordinator and MSF. For the former, it was as a consequence of the duplication of services that was seen when the programmes worked in parallel, while for MSF, it was imperative to ensure the longevity and sustainability of their programme. These different rationale notwithstanding, both state and MSF employees saw the coming together of the two programmes as an extremely positive development. Former MSF employees describe the relationship that developed between the two teams as familial; ‘so there are some people we are still communicating, and we’re so close, we are family’ (iNGO_07), ‘we were just one family’ (iNGO_06). Likewise, government nurses reflecting on the relationship between the two teams described MSF employees in warm and positive ways. Members of the MSF team were described as ‘colleagues’ and as ‘helpful’; ‘they were helping us with transport, everything … I think those people were helping a lot’, and the process of MSF leaving the area, in 2013, as ‘painful’ (DOH_04).

### The handover

MSF's departure from the area was carefully curated and characterised by a handover process that took two and a half years. Both civil servants and MSF acknowledged the time-bound nature of MSF's intervention, with a participant from the Office of the Premier^4^ noting that the ‘government actually has to sustain itself’. Importantly, while the programme was technically handed over to the Provincial DoH, interviews with DoH employees revealed that the Provincial authorities were not involved in the practicalities of the handover. This process was left to the district and sub-district.

MSF described this handover process as ‘a progressive reduction of presence’ (iNGO_01), during which time MSF employees report undertaking a significant amount of capacity building, as well as scaling down the range of services that they had been providing so that the DoH could realistically take over the programme (iNGO_03). This process was planned out using a ‘dashboard’, on which the ‘sequence of the handover was sketched out’ and used to monitor progress (DOH_01; iNGO_03). It involved an integration of the services that the two programmes were offering, skills development and training for DoH staff, and a reduction in the number of farms and staff for outreach visits (iNGO_01). During this process, MSF also reported having to ‘lobby’ the state about the importance of providing care for migrant farm workers, and consequently the need to support the continuation of the Musina Model of Care (iNGO_03). MSF argue that whilst the state employed mobile clinic team was invested in the programme, the District and Provincial level DoH were less willing to redirect resources, and potentially reconsider priorities and policy.

In this process, the CHW component was delinked from the rest of the programme. At the time of MSF's departure, the DoH argued that it was not in a position to take over the management of these workers. Consequently, the management and administration of the programme was handed over to North Star Alliance, an iNGO that provides health services to mobile workers, primarily targeting truck drivers through a series of ‘Roadside Wellness Centres’ (North Star Alliance, 2015).

Reflecting on their departure from Musina and the importance of the handover process, a senior member of MSF in South Africa justified the short term nature of their intervention by explaining how the organisation saw their role as one of innovation rather than ‘system strengthening’:

the aim of our operations isn't to permanently replace provision of services by the national Department of Health. Rather it's to demonstrate a model for some kind of service provision that's replicable and that we’re able to hand over. (iNGO_01)

### After the handover

This research commenced three years after the handover was completed. Although the state-run mobile clinic continues to refer to itself as the Musina Model of Care and to provide ART to migrants living and working on the farms, many of the components of the programme, those which made it particularly innovative, have struggled in the wake of MSF's departure.

In 2017, when interviewed about the success of the programme, a senior MSF employee credited close collaboration between MSF and the DoH, as well as the mobile clinic manager, with the programme's survival and continued success:

I think this is probably one of the reasons that it's survived; so the teams going out were a mixture of MSF staff and DoH staff, so we were able to train and mentor DoH staff. And you can see from [her] (mobile clinic manager) and her team that the model has really survived. And I think it's worth saying that to me it really shows the importance of these kinds of champions. (iNGO_01)

However, this ignores the material advantage that MSF's presence had for the programme. Practically, MSF's involvement brought with it a large team that included individuals whose sole purpose was to organise the logistics of the programme, set up equipment, and drive the clinic from farm to farm, alleviating the nurses of these responsibilities. The driving in particular is deemed strenuous, as the roads between Musina and the surrounding farms are of a poor quality, and the distances between destinations mean that some farms are a two-hour drive from the offices from which the clinic departs each morning. The issue of transport was a key issue to which respondents returned throughout the research. For the DoH nurses, MSF's involvement in and support of the programme was characterised by their provision of vehicles, as one nurse notes ‘when they were here, transport was not a problem. If we didn't have transport, they’d come and pick us up’. And consistent problems with transport serve as a constant reminder of MSF's departure. Whilst the programme has access to three vehicles, Toyota Quantums, these are frequently in need of repairs (echoing findings from mobile clinic programmes in other parts of the country, Stemmet, 2016). Furthermore, several nurses reported that they had clear instructions from the DoH that should they drive in the rain, they remain liable for any mishap or accident on the road.

The provision of care has also become more stressful since MSF's departure as teams are smaller. Additionally, in the beginning of 2018, the mobile clinic manager, who was so strongly praised by MSF, and remains an advocate of the Musina Model of Care*,* was redeployed within the DoH and as such is no longer based in Musina. Her replacement has stopped nurses attending support group meetings over the weekend due to budget constraints. Workers on ART are expected to collect their medication during the clinic's weekly visit, putting additional stress on these visits, or travel to Musina over the weekend.

Finally, it is worth noting how the CHW programme has fared since MSF's departure. Since 2013, the CHW programme has been managed by three different organisations. In the wake of MSF's departure, North Star Alliance volunteered to manage eight of the ten CHWs for a year, whilst the remaining two were taken on by a third organisation – the Treatment Action Campaign – as ‘health mobilisers’. A year later, the programme was handed over to a local NGO, the Centre for Positive Care (CPC), who were able to secure DoH funding to cover stipends for the CHWs. Since the beginning of 2018, the administration of the CHWs has been taken over by the Red Cross, although research participants have reported that there has been very little engagement from the organisation on the ground. No explicit reasons were provided for these frequent changes in management. Meeting minutes and interview data did not evince particular interest or expertise in CHWs among these organisations. Instead, it became clear that these were ad-hoc arrangements put in place to fill in the void created by the departure of a host organisation or its diminishing capacity to manage the programme. For example, at the time of MSF's departure, meeting minutes indicate that North Star Alliance had PEPFAR funding, one of the purposes of which was to ‘undertake a project focused on migrant farm workers in the Musina area’, and as such ‘wishe[d] to assist DoH in sustaining MSF's model of care’. Although many of the farms still have CHWs, some of the original MSF-trained cohorts have left, either in search of better (paid) work or for personal reasons, and on one of the farms in particular farm workers were highly critical of the quality of the CHW's work in more recent years and have become increasingly reliant on the mobile clinic nurses.

Despite these changes, when asked to reflect on whether they thought that the departure of MSF had had an affect on the services that they were able to provide, nurses were quick to respond that they did not. Often adding, for example, ‘we are doing everything they were doing, even though we are lacking there because of transport, we are trying our best’ (DOH_04). Civil servants who were not directly involved in the programme were a bit more critical about how it had fared since MSF's departure, noting that

the level of service delivery really has gone down. Because of, you know, the Department struggling on it's own to provide services. Ja [yes] they are so stretched … transport wise, even personnel … the impression that I got is that they are experiencing more difficulties since the leaving of the Doctors without Borders [MSF]. (OTP_01)

## Discussion and conclusion

In the immediate wake of MSF's departure, the programme was, in many ways, able to continue as it had during MSF's involvement, and mobile clinic staff did not complain about the additional workload. However, without renewed support and the replenishment of resources from the state and other administering organisations, various parts of the programme have started to come undone, bringing its longer term sustainability into question: vehicles need replacing; nurses have left the DoH or Musina and not been replaced; the time that nurses get with patients has been curtailed; and CHWs are unable to provide the support they once did to the mobile clinic.

The stark contrast between working conditions for Mobile Clinic staff during MSF's management and post MSF's departure is understandably the source of much frustration among the health care providers. Research into non-state interventions has often pointed to the frustrations of state actors and the tensions that arise due to the involvement of non-state actors in local contexts (Kapilashrami & McPake, 2013; Kapilashrami & O’Brien, 2012), as well as the differential treatment of local staff from their expatriate counterparts (Pfeiffer, 2003; Redfield, 2012). In this case study, however, since MSF's departure, frustrations were around the inability of the programme to continue in the way it once had, and as such at the lack of support from the state to sustain it. Whilst the material differences in the support offered to the programme during MSF's involvement and since must not be overlooked, part of this frustration is also due to the different ways in which ‘softer’ issues were prioritised by MSF, but are not by the state. MSF prioritised team dynamic and well-being through workshops, supportive training, and building relationships with the DoH team. As such, energies and resources were redirected in ways which nurses felt were worthwhile and important. ‘Softer’ issues have not, however, been prioritised by the DoH. This resonates with work by McIntyre and Klugman (2003, p. 117) which highlights the missed opportunities of not prioritising ‘health worker buy-in’ and ‘recognising and addressing health worker morale issues’ within the public health care system in South Africa. The general lack of attention which is given to ‘softer’ issues within the South African public health system is also central to understanding the lack of workplace trust which Gilson and others have identified in this context (2003, 2006; Gilson, Palmer, & Schneider, 2005) and the difficulties in sustaining the programme. Conceptualisations of programme sustainability centre the importance of the integration of the programme in question into broader structures. For Schell et al. (2013), this is done through the extension of political and financial support to programme and planning. For Shigayeva and Coker (2015), this is achieved through the programme being at the nexus of a series of ‘functional relationships’, with fuller integration within these relationships lending itself to better sustainability. Trust itself ‘is a relational notion’ (Gilson, 2006, p. 360). As such, the lack of integration within ‘functional relationships’ between the local DoH and the DoH at District and Provincial levels points not only to why sustainability in this context is difficult, but also the source of discontent for mobile clinic staff. That being said, it must be recognised that state agencies, in particular those on the ground in Musina, were not consulted in the planning or inception of MSF's activities. Since these agencies have finite capacities and resources and competing demands placed on them, responding to initiatives that were not integrated from the outset with national-level planning, and therefore without allocated funds, pose a significant challenge and distinct burden on ongoing work. This echoes research by Cairney and Kapilashrami (2014) around the effects of PEPFAR and the Global Fund downscaling their activities in Namibia on the local Nambian health system. Authors illustrate how global health insitutions’ demands of rapid scale-up were counterproductive to the sustainability of interventions and the human resources delivering care; and concerns around ownership and political will of state actors emerged only in the wake of a ‘scale down’ of donor funding to the Namibian health sector.

This case study raises two important questions. The first is about what it means to demonstrate an ‘innovative’ model of care if it does not secure the requisite buy-in from the state to sustain the innovation. Emergency humanitarian responses are, by their very nature, not expected to be sustainable, because the understanding is that an emergency is time bound and will come to an end. This intervention, however, like many of MSF's interventions in South Africa, sought to make long-term changes to the mechanisms through which migrant farm workers could access care, with a short-term time commitment from the organisation, and limited assurance that the state would commit to sustaining the intervention. In her assessment of iNGOs who provide funding, Younis raises important questions about ‘the approach of relying on professional nongovernmental organisations (NGOs) that are funded from abroad for achieving social and political change’ (2007, pp. 38, 39). She concludes that the reliance on non-local organisations for funding, and programmatic work in this case, can limit the long term efficacy and sustainability of change as funding may be difficult to replace once they leave. Berlan and Bruno-van Vijfeijken (2013) however argue instead that acknowledging that the interventions of non-local organisations are, by-and-large, ‘time-bound’ and planning for this may be a more sustainable model for iNGOs going forward. Their analysis suggests that planning for an organisation's inevitable departure lends itself to reliance on local actors and consequently the development of more sustainable programmes. However, as this case study shows, within this model the long term commitment of local state and non-state actors is relied upon, but that commitment cannot be assured.

Following on from this, the second question which this case study raises is about what it means to respond to the health of migrants in innovative ways which stem from a response to a ‘crisis’. Vearey (2014) and Vearey et al. (2017) caution against the exceptionalisation of migrants through advocating for migration to be accounted for in general health systems, rather than ad hoc responses to particular migrants and their well-being. Given the way in which the Musina Model of Care (and other interventions which were not the focus of this paper) has fared, however, responses to migration and health in Musina remain bound to the ‘crisis’ of 2007/2008 and the involvement of iNGOs. Targetted responses to improve access to care for certain populations may be necessary. However, in the South African context, short-term initiatives, which exceptionalise such groups, undermine the state's constitutional commitment to the progressive realisation of access to quality and comprehensive health care for all, including migrants. At a global level, these kinds of interventions undermine commitments to and calls for integrated and people-centred health systems, including Universal Health Coverage. In order to uphold these commitments, it is important to develop policy and programmatic responses which acknowledge the realities of migration and mobility. As such, at the handover stage of interventions and programmes, from non-state to state actors, rather than simply focusing on administrative load, differences in priorities amongst stakeholders need to be interrogated.

As organisations like MSF move from more traditional aid interventions, to interventions where they ‘demonstrate a model for some kind of service provision that's replicable and that [they are] able to hand over’ (iNGO_01), understanding whether these interventions lend themselves to longer term structural change and are replicated by the state is important. In this case, five years since the departure of MSF from the area, remnants of the programme remain – importantly, ART is more accessible to farm workers than it was prior to MSF's involvement – as do fond memories of the time that MSF spent in Musina. However, the programme falls significantly short of closing the accessibility gap in health care for remote and migrant populations.

## Notes

1 Responses varied, but this trip reportedly costs between 60 and 150 ZAR (5–12 USD).

2 Important to note is that, in accordance with the *Uniform Patient Free Schedule,* primary health care is free for all in South Africa, and as such all services provided by these two clinic programmes were (and are) provided free of charge.

3 Email correspondence with an MSF employee indicates that this cost the organisation 70,000 ZAR, 4991 USD at the time of writing.

4 The Office of the Premier plays an important role at a Provincial level in South Africa, working with the Provincial Premier and ‘providing coherent strategic leadership and coordination in provincial policy formulation … and implementation’ (The Presidency, 2008, p. 2).
